# A computed tomography radiomics-based model for predicting osteoporosis after breast cancer treatment

**DOI:** 10.1007/s13246-023-01360-2

**Published:** 2024-01-08

**Authors:** Yu-Hsuan Lai, Yi-Shan Tsai, Pei-Fang Su, Chung-I Li, Helen H. W. Chen

**Affiliations:** 1 Institute of Clinical Medicine, College of Medicine, National Cheng Kung University, Tainan, Taiwan; 2 Department of Radiation Oncology, National Cheng Kung University Hospital, College of Medicine, National Cheng Kung University, Tainan, Taiwan; 3 Department of Oncology, National Cheng Kung University Hospital, College of Medicine, National Cheng Kung University, No. 138 Sheng-Li Rd, Tainan, 704302 Taiwan; 4 Clinical Innovation and Research Center, National Cheng Kung University Hospital, College of Medicine, National Cheng Kung University, Tainan, Taiwan; 5 Department of Medical Imaging, National Cheng Kung University Hospital, College of Medicine, National Cheng Kung University, Tainan, Taiwan; 6 Department of Statistics, National Cheng Kung University, Tainan, Taiwan

**Keywords:** Breast cancer, Cancer treatment-induced bone loss, Computed tomography, Osteoporosis, Predictive modelling, Radiomics

## Abstract

Many treatments against breast cancer decrease the level of estrogen in blood, resulting in bone loss, osteoporosis and fragility fractures in breast cancer patients. This retrospective study aimed to evaluate a novel opportunistic screening for cancer treatment-induced bone loss (CTIBL) in breast cancer patients using CT radiomics. Between 2011 and 2021, a total of 412 female breast cancer patients who received treatment and were followed up in our institution, had post-treatment dual-energy X-ray absorptiometry (DXA) examination of the lumbar vertebrae and had post-treatment chest CT scan that encompassed the L1 vertebra, were included in this study. Results indicated that the T-score of L1 vertebra had a strongly positive correlation with the average T-score of L1–L4 vertebrae derived from DXA (r = 0.91, p < 0.05). On multivariable analysis, four clinical variables (age, body weight, menopause status, aromatase inhibitor exposure duration) and three radiomic features extracted from the region of interest of L1 vertebra (original_firstorder_RootMeanSquared, wavelet.HH_glcm_InverseVariance, and wavelet.LL_glcm_MCC) were selected for building predictive models of L1 T-score and bone health. The predictive model combining clinical and radiomic features showed the greatest adjusted R^2^ value (0.557), sensitivity (83.6%), specificity (74.2%) and total accuracy (79.4%) compared to models that relied solely on clinical data, radiomic features, or Hounsfield units. In conclusion, the clinical-radiomic predictive model may be used as an opportunistic screening tool for early identification of breast cancer survivors at high risk of CTIBL based on non-contrast CT images of the L1 vertebra, thereby facilitating early intervention for osteoporosis.

**Supplementary Information:**

The online version contains supplementary material available at 10.1007/s13246-023-01360-2.

## Introduction

Globally, 1.7 million women are diagnosed with breast cancer every year, with overall survival estimated to be greater than 80% in high-income countries [1]. In Taiwan, breast cancer is the most common cancer among women, and the incidence rate is increasing [2]. Breast cancer therapy, including chemotherapy, endocrine therapy and oophorectomy, is associated with increased fracture risk due to deterioration of bone quantity and quality caused by decreased blood level of estrogen [3–5]. This cancer treatment-induced bone loss (CTIBL) represents the most common long-term adverse event in breast cancer survivors and is responsible for osteoporosis and fragility fractures [3]. Therefore, the early diagnosis of osteoporosis and prevention of bone mineral loss are important to further support the quality of life and life expectancy of breast cancer survivors [6].

During diagnosis and treatment for breast cancer, thoracic computed tomography (CT) is widely used to monitor disease status and treatment efficacy [7]. Nonetheless, dual-energy X-ray absorptiometry (DXA) examination, the gold-standard method for assessing bone mineral density (BMD), may not always be a component of the post-treatment regimen [8], despite recommendations for BMD assessment and regular monitoring [9, 10]. In contrast, the regular use of CT scans in the patients who do not undergo DXA examination has been used for opportunistic osteoporosis screening and evaluation of bone loss [11, 12]. CT attenuation values in Hounsfield units (HU) of the lumbar vertebra calculated from the images were applied to opportunistically detect patients at high risk of osteoporosis, thereby avoiding DXA-related radiation exposure or costs [11]. However, current imaging modalities, such as DXA and CT HU, only measure bone density but fail to assess bone quality, another important aspect of bone strength which takes bone architecture, turnover, damage accumulation, and mineralization into account. In contrast, radiomics, the high-throughput extraction of large amounts of image features from radiographic images, has recently attracted much attention [13]. Although extensively used in clinical oncology [14], this approach has been limitedly explored for the detection and diagnosis of bone diseases [15]. Radiomic features, ranging from pixel density and arrangement to textures, intensity and wavelet features, can provide a comprehensive evaluation of both bone quantity and quality.

The present study proposed novel CT radiomics-based opportunistic screening methods for detecting CTIBL in breast cancer patients. Because the chest CT scan routinely extended from thoracic inlet to upper abdomen, the L1 vertebra was always encompassed in the chest CT scan. Therefore, we hypothesized that the CT image of the L1 vertebra may be useful for the development of predictive model for treatment-induced osteoporosis in breast cancer survivors. To test this hypothesis, this study aimed: (1) to examine whether L1 T-score was significantly correlated with the L1–L4 mean T-score (gold standard); (2) to extract radiomic features from axial non-contrast CT images of the L1 vertebra for each patient and (3) to apply radiomic features and/or clinical data to build predictive models for L1 T-score and bone health.

## Methods

### Study participants

This retrospective study was approved by the institutional review board of the National Cheng Kung University Hospital (NCKUH). Female patients who were diagnosed with breast cancer and received a complete course of treatment and follow-up between 2011 and 2021 were initially selected. Inclusion criteria were: (i) patients who underwent post-treatment thoracic CT scan and had unenhanced CT images that showed L1 vertebra; (ii) patients who underwent post-treatment DXA scan of the lumbar spine (L1 to L4 vertebrae) and (iii) the time interval between CT scan and DXA scan was less than 1 year. Exclusion criteria were: (i) patients with lumbar scoliosis, lumbar compression fractures, or radiodense osteophytosis in the lumbar vertebrae and (ii) patients with a history of lumbar surgery.

### Study variables

Subjects’ demographic information and clinical characteristics were recorded from medical charts, such as age, body weight, menopause status, tobacco and alcohol use, exercise habits, comorbidities related to the fracture risk assessment tool (FRAX), T-scores of the lumbar spine derived from DXA and systemic treatments for breast cancer such as chemotherapy, endocrine therapy and targeted therapy. Commonly used endocrine therapy for breast cancer included tamoxifen (TAM) for premenopausal women to block estrogen receptors and aromatase inhibitor (AI) for postmenopausal women to lower estrogen levels. The exposure duration of endocrine therapy was also collected. The BMD was measured with GE Lunar Prodigy and the newer GE Lunar iDXA densitometers (both from GE Healthcare Lunar, Madison, WI, USA). The T-score was a standard deviation showing how much the measured bone density differed from the bone density of a healthy young adult. According to the World Health Organization (WHO), osteoporosis, osteopenia and normal subjects were defined based on mean lumbar spine (L1–L4) T-score: osteoporosis (T-score ≤ -2.5), osteopenia (-2.5 < T-score < -1.0) and normal (T-score ≥ -1.0) [16].

### Radiomic feature extraction on non-contrast CT images

A total of 5 CT scanners were used for non-contrast chest CT scanning between 2011 and 2021. Of them, 2 scanners were purchased from GE Healthcare (Chicago, IL, USA) and 3 scanners were obtained from Siemens Healthineers (Erlangen, Germany). Additional manufacturer information is summarized in Supplementary Table S1. All non-contrast CT images were collected using Digital Imaging and Communications in Medicine (DICOM) format and retrieved from the picture archiving and communication system (PACS) at NCKUH. The DICOM images were saved into Neuroimaging Informatics Technology Initiative file format, and the image segmentation was performed using a self-invented image-labeling tool running on INFINITE PACS 3.0 as previously described [17]. Radiomic features were calculated using wavelet and Laplacian of Gaussian with sigma filters.

The region of interest (ROI) on axial non-contrast CT image of the L1 vertebra was manually labeled by two radiologists who were blinded to the clinicopathological details of patients. The ROI was drawn on the mid-vertebral level of the L1 vertebral body to avoid cortical bone and the basivertebral vein (Supplementary Fig. S1), as previously described [18]. Quantitative radiomic features were then extracted from the ROIs using the PyRadiomics v3.0.1 package [19]. All steps of ROI labeling were repeated one month later by the junior radiologist to test the intra-observer variability [20].

Radiomic features were defined according to the PyRadiomics library [17]. Radiomic features for each patient were extracted three times by two radiologists (twice from the junior radiologist and once from the senior radiologist). A total of 477 radiomic features were initially extracted from the CT images of the patient population. According to the previously published criterion, the intraclass correlation coefficients (ICC) < 0.75 [20], 28 radiomic features were excluded. Therefore, 449 radiomic features with good to excellent ICC were retained for statistical analysis.

**Fig. 1:**
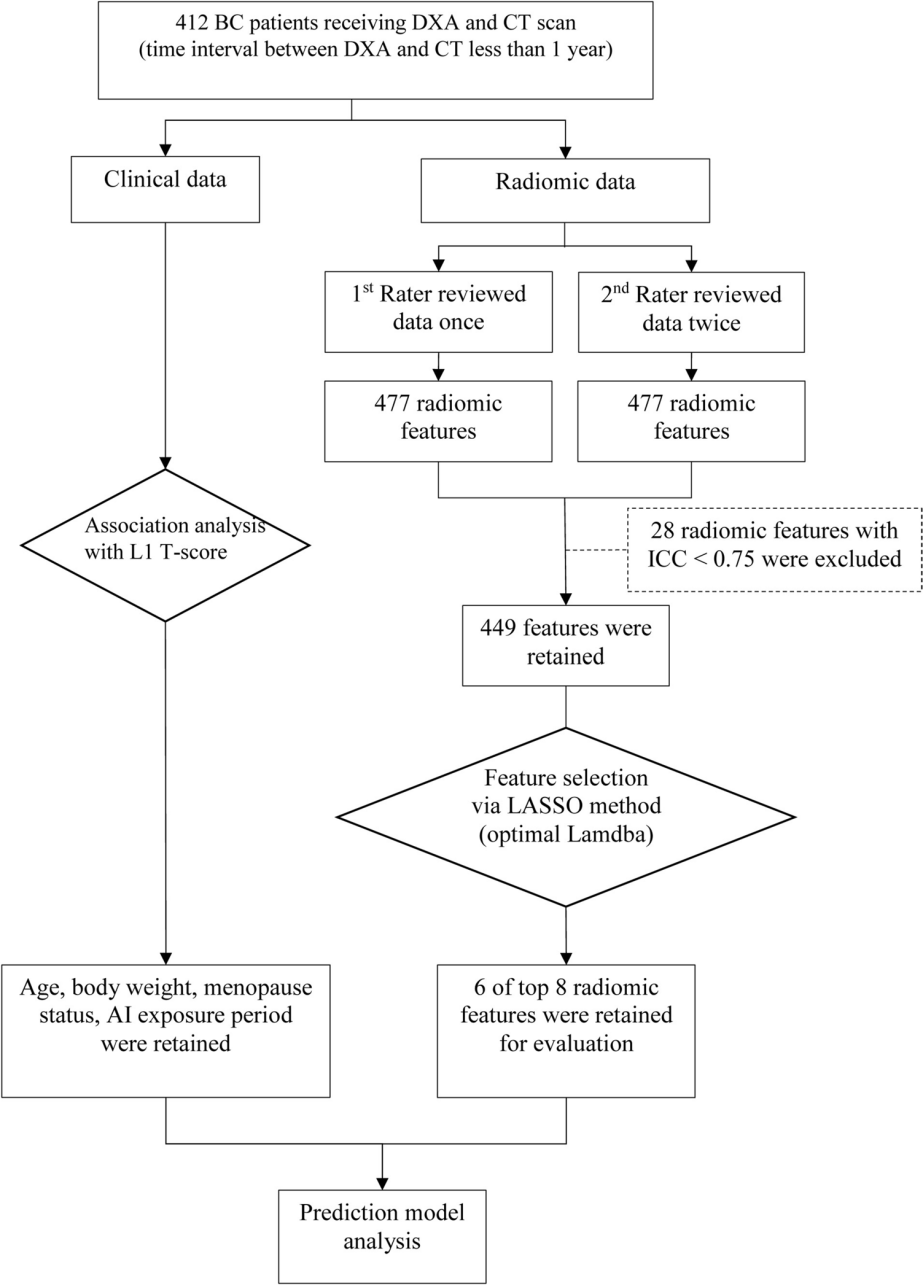
Flow diagram of data processing and analysis

Subsequently, 8 out of 449 radiomic features with optimal lambda values were selected by the least absolute shrinkage and selection operator (LASSO) regression analysis (Supplementary Table S2). However, because of high correlation with each other, two radiomic features were further excluded. As a result, only 6 radiomic features were retained for the final statistical analysis (Supplementary Table S2). Data processing and analysis for the present study is shown in Fig. 1.

### Statistical analysis

Data are presented as mean ± standard deviation (SD) for continuous variables, and n (%) for categorical variables. Spearman correlation analysis was performed to evaluate the association of L1 T-score and the average T-score of L1–L4 vertebrae derived from DXA. Results are presented in a scatter plot with the coefficient of correlation (r) and *p*-value. Furthermore, associations between L1 T-score and clinical data were evaluated using univariable linear regression analysis, and the results are presented as ß with 95% confidence intervals (95% CI) and *p*-values. Significant variables (*p* < 0.05) were included in the subsequent predictive model analysis.

After extraction of radiomic features, four steps were sequentially conducted to select radiomic features for building predictive models for bone mineral loss. First, the useful radiomic features were preliminarily selected using LASSO regression, in which the optimal Lamdba value was set based on minimum of mean cross-validation error. Second, because radiomic features were sensitive to variation across scanners and acquisition protocols, normalization of radiomic features was required [21, 22]. Therefore, each radiomic feature was normalized as the range from − 1 to 1. The normalized radiomic features were expressed as mean ± SD for the subsequent analysis. Third, the radiomic features that were highly correlated with others were excluded, and the remaining radiomic features were subjected to univariable and multivariable linear regression analyses to evaluate their associations with L1 T-score. Finally, the radiomic features with *p* < 0.05 identified by either univariable or multivariable analysis were retained as candidate radiomic features for building predictive models with or without clinical data. In addition, to determine whether a linear model was appropriate and to examine the assumption that the statistical model played a vital role, the residual analyses, including checking normality, independence, and testing homogeneity of variance, were performed [23]. The results indicated that the statistical model fitted the data well.

Based on the multivariable linear regression results, three models for predicting L1 T-score were built, including Model I (clinical data), Model II (radiomic data) and Model III (both clinical and radiomic data). The results of predictive models were presented as ß with 95% CI and *p*-values, as well as adjusted R^2^ statistics for each model. In order to evaluate the diagnostic accuracy of predictive models, a confusion matrix with a total accuracy, sensitivity, and specificity was calculated based on the golden standard for diagnosis of osteoporosis. Study variables were assessed using a two-tailed test and a *p*-value < 0.05 was considered statistically significant. All statistical analyses were performed using R Statistical software (version 4.0.2; R Foundation for Statistical Computing, Vienna, Austria).

## Results

A total of 412 breast cancer patients receiving post-treatment DXA examination and thoracic CT scan were included in the study. Clinical data and CT-based radiomic features were collected from all 412 patients.

**Fig. 2:**
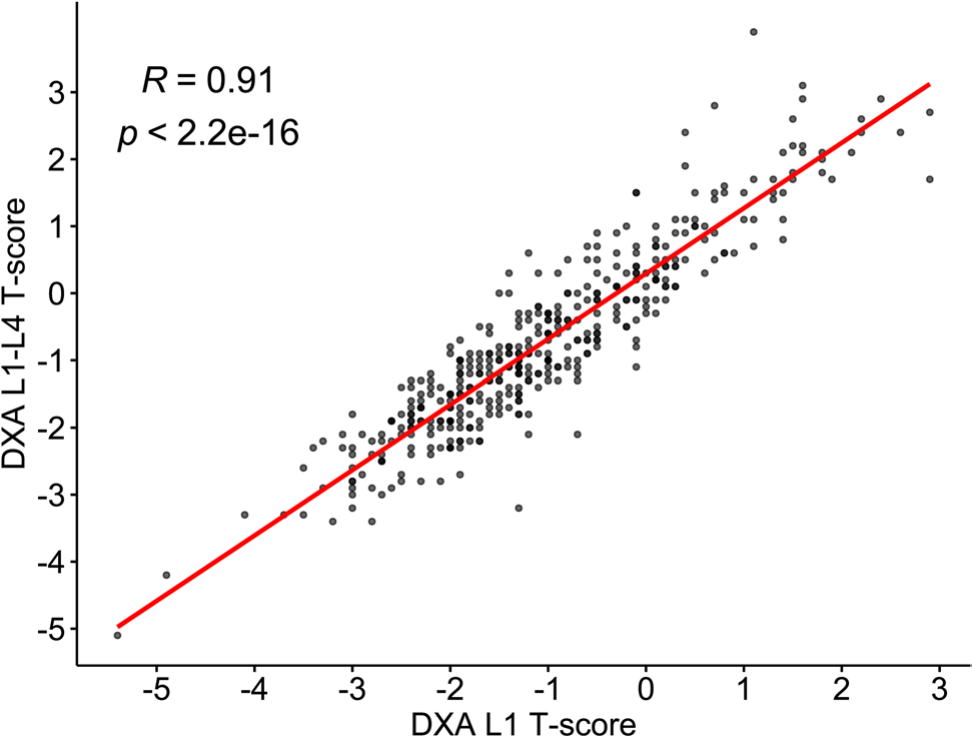
Correlation analysis between T-score of L1 vertebra and the average T-score of L1–L4 vertebrae derived from DXA

Patient demographics and baseline clinical characteristics, including WHO FRAX parameters, systemic treatments, and bone health examination (T-score of L1), are summarized in Table 1. The mean T-score of L1 vertebra for all patients was − 1.05 ± 1.28. The univariable analysis revealed that the L1 T-score was significantly associated with age, weight, post menopause, smoking status, TAM exposure, and AI exposure years (all *p* < 0.05; Table 1). The correlation analysis showed a high correlation between the T-score of L1 vertebra and the average T-score of L1–L4 vertebrae derived from DXA (r = 0.91, *p* < 0.05; Fig. 2).

**Table 1:** Univariable analysis for associations between demographic and clinical characteristics and L1 T-score (N = 412)

Variables	Total^a^	Univariable analysis^b^	
	(N = 412)	ß (95% CI)	*p*-value
Demographics			
Age (years)	61.5 ± 10.1	− 0.037 (− 0.049, − 0.025)	** < 0.001**
Weight (kg)	58.7 ± 10.0	0.047 (0.036, 0.059)	** < 0.001**
Post menopause	383 (93.0%)	− 1.085 (− 1.560, − 0.610)	** < 0.001**
The WHO FRAX			
Smoking	6 (1.5%)	− 1.299 (− 2.330, − 0.268)	**0.014**
Alcohol	2 (0.5%)	− 1.152 (− 2.938, 0.634)	0.206
Exercise (weekly frequency)			
< 1	273 (66.3%)	1	
1–2	63 (15.3%)	0.213 (− 0.140, 0.566)	0.236
3–5	59 (14.3%)	0.151 (− 0.212, 0.513)	0.415
6–7	17 (4.1%)	0.057 (− 0.574, 0.688)	0.859
Glucocorticoid use	1 (0.2%)	− 1.250 (− 3.775, 1.275)	0.331
Rheumatoid arthritis	2 (0.5%)	0.104 (− 1.686, 1.894)	0.909
Hyperthyroidism	2 (0.5%)	− 0.198 (− 1.987, 1.592)	0.828
Chronic liver disease	15 (3.6%)	− 0.035 (− 0.699, 0.630)	0.919
Fracture history	17 (4.1)	− 0.227 (− 0.852, 0.399)	0.447
Systemic treatments			
Chemotherapy	267 (64.8%)	0.006 (− 0.255, 0.266)	0.964
Endocrine therapy	360 (87.4%)	0.084 (− 0.290, 0.459)	0.659
TAM exposure	151 (36.7%)	0.361 (0.106, 0.617)	**0.006**
TAM exposure years	0.57 ± 1.33	0.050 (− 0.044, 0.144)	0.295
AI exposure	303 (73.5%)	− 0.257 (− 0.538, 0.024)	0.073
AI exposure years	1.13 ± 1.72	− 0.086 (− 0.159, − 0.015)	**0.019**
Targeted therapy	54 (13.1%)	− 0.209 (− 0.577, 0.159)	0.264
Bone health examination			
L1 T-score	− 1.05 ± 1.28		

*WHO FRAX* World Health Organization fracture risk assessment tool, *TAM* tamoxifen, *AI* aromatase inhibitor

Bold *p*-value indicated significance (p < 0.05)

^a^Data were presented as Mean ± SD; or n (%)

^b^The results were presented as ß with 95% confidence intervals (95% CI) and *p*-value via univariate liner regression model analysis

The six normalized CT-based radiomic features were then subjected to univariable and multivariable analysis. Univariable analysis revealed that the L1 T-score was significantly associated with all the six radiomic features, including original_firstorder_RootMeanSquared (ß = 0.825), wavelet.LH_glcm_IverseVariance (ß = 0.222), wavelet.HL_glcm_IverseVariance (ß = 0.228), wavelet.HH_glcm_InverseVariance (ß = 0.302), wavelet.HH_glrlm_RunEntropy (ß = − 0.164), and wavelet.LL_glcm_MCC (ß = 0.141) (All *p* < 0.05; Table 2). Multivariable analysis revealed that only three radiomic features, including original_firstorder_RootMeanSquared (ß = 0.775), wavelet.HH_glcm_InverseVariance (ß = 0.218), and wavelet.LL_glcm_MCC (ß = 0.197), remained significantly associated with the L1 T-score (all *p* < 0.001; Table 2). Hence, only the last 3 radiomic features were subjected to the subsequent multivariable analysis and the predictive model building process.

**Table 2:** Associations between L1 T-score and radiomic features

Radiomic features	Univariable analysis		Multivariable analysis	
	ß (95% CI)	*p*-value	ß (95% CI)	*p*-value
Original series				
original_firstorder_RootMeanSquared	0.825 (0.730, 0.921)	** < 0.001**	0.775 (0.681, 0.868)	** < 0.001**
Wavelet series				
wavelet.LH_glcm_IverseVariance	0.222 (0.099, 0.344)	** < 0.001**	0.063 (− 0.063, 0.188)	0.326
wavelet.HL_glcm_IverseVariance	0.228 (0.105, 0.351)	** < 0.001**	0.056 (− 0.067, 0.179)	0.372
wavelet.HH_glcm_InverseVariance	0.302 (0.181, 0.423)	** < 0.001**	0.218 (0.100, 0.335)	** < 0.001**
wavelet.HH_glrlm_RunEntropy	− 0.164 (− 0.288, − 0.041)	**0.009**	0.009 (− 0.103, 0.122)	0.869
wavelet.LL_glcm_MCC	0.141 (0.017, 0.265)	**0.026**	0.197 (0.089, 0.305)	** < 0.001**

Univariate linear regression analysis was conducted, and the results were presented as coefficient (ß) with corresponding 95% confidence intervals (95% CI) and *p*-value. For multivariable analysis, all 8 radiomic features were proceeded via multivariable linear regression analysis and the results were presented as ß with 95% CI and *p*-value

Bold p-value indicates significantly (*p* < 0.05)

Results of multivariable linear regression analyses of three models for predicting L1 T-score are shown in Table 3. The model I used age, weight, post menopause, and AI exposure, whereas model II considered 3 radiomic features. Model III utilized both clinical data and radiomic features. The adjusted R^2^ values were 0.229, 0.452 and 0.557 for model I, II and III, respectively (Table 3), suggesting a better performance of prediction using model III. Based on the calculated coefficients, the accuracy of L1 T-score prediction in model III increased with five variables, including age, weight, original_firstorder_RootMeanSquared, wavelet.HH_glcm_InverseVariance and wavelet.LL_glcm_MCC. In contrast, L1 T-score prediction in model III decreased with post menopause and AI exposure.

**Table 3:** Multivariable models for predicting L1 T-score based on clinical data, radiomic data, or combination of clinical + radiomic data

Characteristic	Model I: clinical data (adjusted R^2^ = 0.229)		Model II: radiomic data (adjusted R^2^ = 0.452)		Model III: clinical + radiomic data (adjusted R^2^ = 0.557)	
	ß (95% CI)	*p*-value	ß (95% CI)	*p*-value	ß (95% CI)	*p*-value
Age (yrs)	− 0.032 (− 0.043, − 0.020)	** < 0.001**			0.019 (0.008, 0.031)	** < 0.001**
Weight (kg)	0.047 (0.036, 0.058)	** < 0.001**			0.041 (0.032, 0.050)	** < 0.001**
Post menopause (vs. No)	− 0.543 (− 1.013, − 0.074)	**0.023**			− 0.576 (− 0.933, − 0.220)	**0.002**
AI exposure (yrs)	− 0.031 (− 0.095, 0.034)	0.350			− 0.031 (− 0.081, 0.019)	0.222
original_firstorder_RootMeanSquared			0.776 (0.682, 0.870)	** < 0.001**	0.859 (0.750, 0.970)	** < 0.001**
wavelet.HH_glcm_InverseVariance			0.270 (0.168, 0.371)	** < 0.001**	0.073 (− 0.027, 0.173)	0.150
wavelet.LL_glcm_MCC			0.199 (0.099, 0.300)	** < 0.001**	0.181 (0.089, 0.274)	** < 0.001**

The results were presented as ß) with 95% CI and *p*-value

Bold *p*-value indicated significance (*p* < 0.05)

Bone health classified into two categories based on L1 T-score, normal (T-score ≥  − 1.0) and bone loss (T-score <  − 1.0), followed by an evaluation of the diagnostic performance of three models for predicting bone health (Table 4). Among three predictive models, model III (clinical-radiomic model) had the greatest sensitivity (83.6%), specificity (74.2%) and total accuracy (79.4%), suggesting the superiority of model III over the other two models that relied solely on clinical data or radiomic features in predicting bone heath.

**Table 4:** Diagnostic performance of models for predicting bone health

Predicted bone health	Model I: clinical data (%)	Model II radiomic data (%)	Model III clinical + radiomic data (%)
Sensitivity	73.0	77.9	83.6
Specificity	61.3	72.0	74.2
Total accuracy	67.7	75.2	79.4

Bone health was categorized as normal (T-score ≥ -1) and bone loss (T-score < -1), Sensitivity (True positive rate) = Truly predicted bone loss/truly observed bone loss, Specificity (True negative rate) = Truly predicted normal bone health/truly observed normal bone health, Total accuracy = (True positive + True negative)/Total population

In the present study, 412 patients with a timeframe of less than 1 year between CT scan and DXA scan were included, consisting of 107 patients with 0–30 days, 93 patients with 31–90 days, 88 patients with 91–180 days, and 124 patients with 181–365 days. To evaluate the effects of time interval on predictive performance, the patients were stratified by the time interval between DXA scan and CT scan in two ways. In the first attempt, DEXA-CT.90, patients were divided into two groups using a cut-off of 90 days (0–90 days vs. 91–365 days). In the second attempt, DEXA-CT.30, patients were classified into two groups using a cut-off of 30 days (0–30 days vs. 31–365 days). For predicting T-score, the univariable linear regression analysis revealed that there were no significant differences in predictive performance between 0–90 days and 91–365 days, as well as between 0–30 days and 31–365 days (Table 5). For predicting bone health, the univariable logistic regression analysis also indicated no significant differences in predictive performance between 0–90 days and 91–365 days, as well as between 0–30 days and 31–365 days (Table 5). The results indicated no significant differences in performance of predictive models no matter the shorter or longer DXA-CT intervals.

**Table 5:** Performance comparison between prediction models with different time intervals between CT and DAX scans. Univariable linear regression models for predicting L1 T-score. Univariable logistic regression models for predicting L1 bone health

Time period	N	n (%)	Coef	SE	95% CI	*p*-value
DEXA_CT.90	412					
0–90 days		200 (48.54%)				
91–365 days		212 (51.46%)	0.102	0.127	− 0.146, 0.351	0.420
DEXA_CT.30	412					
0–30 days		107 (25.97%)				
31–365 days		305 (74.03%)	− 0.128	0.144	− 0.411, 0.156	0.376

Time period	N	L1 bone health		Estimate	SE	OR (95% CI)	*p*-value
		Normal, n (%)	Abnormal, n (%)				
DEXA_CT.90	412						
0–90 days		88 (47.3%)	112 (49.6%)				
91–365 days		98 (52.7%)	114 (50.4%)	− 0.09	0.20	0.91 (0.62, 1.35)	0.650
DEXA_CT.30	412						
0–30 days		53 (28.5%)	54 (23.9%)				
31–365 days		133 (71.5%)	172 (76.15)	0.24	0.23	1.27 (0.82, 1.98)	0.290

Furthermore, we conducted linear regression analysis to compare performance between the Hounsfield model and three predictive models for predicting L1 T-score (Supplementary Table S3). The results revealed that the adjusted R^2^ values were 0.411, 0.452 and 0.557 for the Hounsfield model, radiomic model (model II) and clinical-radiomic model (model III), respectively. Besides, model III had the best predictive performance for bone health among these models, suggesting an improved ability to predict L1 T-score and bone health using clinical-radiomic model rather than the Hounsfield model.

## Discussion

This retrospective study utilized clinical and CT-based radiomic features collected from patients who received post-treatment CT and DXA examinations to build the predictive models for bone loss, in order to early identify breast cancer survivors at high risk of osteoporosis after treatment. A high correlation between the T-score of L1 vertebra and the average T-score of L1–L4 vertebrae derived from DXA was observed. Multivariable analysis revealed that L1 T-score was significantly associated with three radiomic features, including original_firstorder_RootMeanSquared, wavelet.HH_glcm_InverseVariance, and wavelet.LL_glcm_MCC, which were subjected to the predictive model building process. Compared to the clinical data- or radiomic feature-based predictive models, the predictive model combining clinical data and radiomic features had the highest adjusted R^2^ value, suggesting a better performance in predicting L1 T-score. Consistently, the model III (clinical-radiomic model) had the greatest sensitivity, specificity, and total accuracy in predicting bone loss. Moreover, the current findings suggested that clinical-radiomic model had a better predictive performance than the Hounsfield model, and the length of time interval between DXA and CT scans did not affect performance of predictive models.

With DXA as reference, opportunistic screening for osteoporosis using CT attenuation values derived from the CT scan has been reported [8, 11, 21, 22]. Notably, Pickhardt et al. assessed BMD using CT and DXA for identifying osteoporosis, with an emphasis on L1 measures [24]. Recently, Park et al. found that compared to DXA, CT attenuation values can be used for predicting osteoporosis and discriminating incidental fracture risk in breast cancer patients [8]. A meta-analysis by Ahern et al. demonstrated that the HU was a clinically useful tool to aide in the diagnosis of osteoporosis; however, determining the optimal HU cut-off was troublesome [25]. In contrast, the present retrospective study developed a novel approach with the high throughput radiomic features for opportunistic screening for bone loss in breast cancer survivors after treatment, providing a comprehensive evaluation of bone quantity and quality, and no optimal cut-off values of radiomic features were required.

In the present study, the positive correlation between L1 T-score and the average T-score of L1–L4 measured using DXA was first demonstrated, and the predicted L1 T-score was then used for predicting bone loss. CT radiomics-based predictive models were subsequently built. A recent study demonstrated that radiomics analysis based on lumbar spine CT scans was an effective method to screen for osteoporosis, with a greater net benefit than the Hounsfield model [26]. In agreement with this finding, the present study found that sensitivity, specificity and accuracy of Model III (clinical and radiomic data) were higher than those of the Hounsfield model, demonstrating the effectiveness of the clinical-radiomic predictive model for the early detection of patients with bone loss after breast cancer treatment, without additional radiation exposure or cost due to DXA.

Endocrine therapy is a standard treatment for hormone receptor-positive breast cancer and is associated with a significant reduction in disease recurrence and improvement in overall survival [27], but endocrine therapy is also associated with an increased risk of osteoporosis and osteoporotic fracture, particularly among patients receiving AI therapy [28]. The present study found that the L1 T-score was positively associated with TAM usage and negatively associated with AI exposure duration, consistent with literature about the impact of endocrine therapy on bone loss [26, 28]. A Canadian retrospective study found a reduced risk of osteoporotic fracture associated with TAM usage over time in postmenopausal patients with early-stage breast cancer [28]. A prospective substudy of the Anastrozole, Tamoxifen, Alone or in Combination (ATAC) trial reported that anastrozole was significantly associated with accelerated bone loss over time in postmenopausal women with breast cancer [29]. Detailed clinical data with endocrine exposure duration included made our results more convincing.

In oncology, radiomics is primarily used for the non-invasive estimation of a clinical diagnosis or prognosis [14]. However, Jiang et al. recently aimed to validate a radiomic signature based on CT scans to screen for lumbar spine osteoporosis [26]. The signature model demonstrated excellent prediction performance for osteoporosis, suggesting that this methodology may facilitate surgical decision-making without additional medical costs and radiation exposure [30]. The above result is consistent with the current study, as well as with other recent publications utilizing MRI or CT scans, supporting radiomic models based on lumbar spine images to detect osteoporosis [30, 31].

Three key radiomic features associated with bone loss identified in the current study were original firstorder_RootMeanSquared, wavelet-HH_glcm_InverseVariance and wavelet-LL_glcm_MCC. RootMeanSquared (RMS) is defined as the square-root of the mean of all the squared intensity values, which indicates the magnitude of the image values. In the multivariate analysis, the beta value of this radiomic feature was 0.775, so RMS was positively correlated with the L1 T-score. The greater the RMS (the greater magnitude of the image values), the higher the L1 T-score (the higher the bone density, or the more solid and complete the bone structure). The larger the InverseVariance value, the smaller the variance and vice versa. In the multivariate analysis, the beta value of this radiomic feature was 0.218, so InverseVariance was positively correlated with the L1 T-score. The larger the InverseVariance (the smaller the variance), the higher the T-score (the higher the bone density, or the more solid and complete the bone structure). The maximal correlation coefficient (MCC) is a measure of complexity of the texture. In the multivariate analysis, the beta value of this radiomic feature was 0.197, so MCC was positively correlated with the L1 T-score. The healthy bone with intact cortex and medulla has a higher MCC value. In contrast, the bone with osteopenia (or osteoporosis) has a lower MCC value. These radiomic features are clinically interpretable and can provide a deep look at the bone quality.

To provide a comprehensive view of the radiomic model’s performance a β with 95% CI and R^2^ (coefficient of determination) were used, metrics that are widely used to evaluate linear regression models. The β coefficient measures the strength of the linear relationship between the predictor and response variables, and the 95% CI provides an estimate of the confidence of the model’s predictions. The R^2^ statistic measures the proportion of variance in the response variable that is explained by the predictor variables and it is a useful measure for assessing the overall accuracy of the model [32]. Worth noting, area under the curve (AUC) is not typically used for linear regression models, and the current study primarily focused on the prediction of L1 T-score by linear regression models.

Strengths of the current study include the pilot construction of a radiomics-based model for predicting L1 T-score in breast cancer patients, opportunistic use of post-treatment thoracic CT for screening CTIBL and the speed of the screening tool utilized. The inherited limitation of this retrospective, single-institution pilot study was small sample size. Thus, all data were used to build predictive models in the present study. Although the validity of predictive models was determined by residual analysis, the generalization of current findings is difficult due to the lack of additional dataset (test dataset). Therefore, large-scale multicenter studies are warranted to evaluate generalization of the clinical-radiomic predictive model as an opportunistic screening tool. In addition, the present study demonstrated that no significant differences in performance of predictive models no matter the shorter or longer DXA-CT intervals. However, the possibility that small sample sizes lead to non-significant statistical results cannot be ruled out. Hence, to specifically exploring the effect of the longer time interval on performance of clinical-radiomic model, large-scale multi-center studies are warranted.

## Conclusion

DXA is not routinely performed during breast cancer treatment in clinical practice, so treatment-induced osteoporosis in breast cancer survivors might be overlooked without prompt management to prevent further bone loss and possible osteoporotic fracture. This study developed a novel clinical-radiomic model for predicting L1 T-score and bone health with superior predictive performance than the Hounsfield model. The proposed clinical-radiomic model may be used as an opportunistic screening tool to help identify breast cancer survivors at high risk of osteoporosis, without DXA-related medical costs and radiation exposure, to achieve early detection and intervention.

### Supplementary Information

Below is the link to the electronic supplementary material.

Supplementary file1 (TIFF 569 kb)—Supplementary Fig. S1 The axial unenhanced CT image of the L1 vertebra was used for image segmentation. The region of interest (ROI; yellow circle) was drawn on the mid-vertebral level of the L1 vertebral body avoiding cortical bone and basivertebral vein.

Supplementary file2 (DOCX 23 kb)

## Data Availability

All data generated or analyzed during this study are included in this published article and its supplementary information files.
