# Supplementary figures and images for: A computed tomography radiomics-based model for predicting osteoporosis after breast cancer treatment
Source: Phys Eng Sci Med. 2024 Jan 8;47(1):239–48. doi: 10.1007/s13246-023-01360-2 (PMC10963549; doi:10.1007/s13246-023-01360-2)

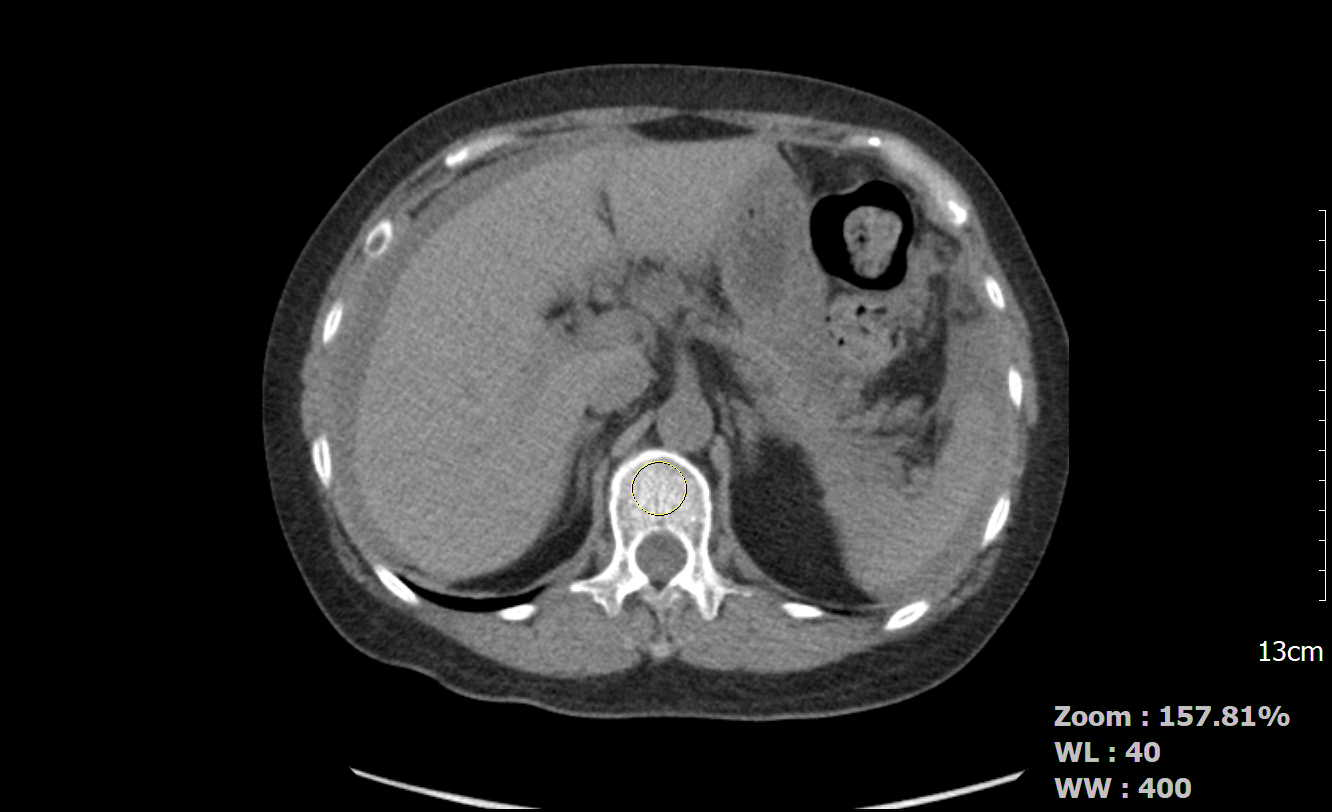

Supplement: Supplementary file 1 — Supplementary file1 (TIFF 569 kb)—Supplementary Fig. S1 The axial unenhanced CT image of the L1 vertebra was used for image segmentation. The region of interest (ROI; yellow circle) was drawn on the mid-vertebral level of the L1 vertebral body avoiding cortical bone and basivertebral vein. [file 13246_2023_1360_MOESM1_ESM.tiff]
