# Supplementary material for: A computed tomography radiomics-based model for predicting osteoporosis after breast cancer treatment
Source: Phys Eng Sci Med. 2024 Jan 8;47(1):239–48. doi: 10.1007/s13246-023-01360-2 (PMC10963549; doi:10.1007/s13246-023-01360-2)
Supplement: Supplementary file 2 — Supplementary file2 (DOCX 23 kb) [file 13246_2023_1360_MOESM2_ESM.docx]

**Supplementary Table S1**. The manufacturer, model, and protocol of CT scanners used in this study.

| CT make and model | GE LightSpeed **RT4** | Siemens Sensation 16 | | GE Optima CT 660 | | Siemens Definition AS | | Siemens Definition Flash | |
| --- | --- | --- | --- | --- | --- | --- | --- | --- | --- |
| Specific anatomic region | Chest | Abdomen | Chest | Abdomen | Chest | Abdomen | Chest | Abdomen | Chest |
| Low kilovoltage | 120 | 120 | 120 | 120 | 120 | 120 | 120 | 120/260 | 120 |
| Effective mAs | 200 | 200 | 130 | 490 | 310 | 200 | 110 | 260 | 110 |
| Rotation time (sec) | 1 | 0.5 | 0.5 | 0.5 | 0.5 | 0.5 | 0.5 | 0.5 | 0.5 |
| Detector | 4 | 16 | 16 | 64 | 64 | 32 | 32 | 128 | 128 |
| Slice | 4 | 16 | 16 | 128 | 128 | 64 | 64 | 256 | 256 |
| Detector Collimation (mm) | 5 | 1.5 | 1.5 | 0.625 | 0.625 | 0.6 | 0.6 | 0.6 | 0.6 |
| Slice Thickness (mm) | 5.0 | 5.0 | 5.0 | 5.0 | 5.0 | 5.0 | 5.0 | 5.0 | 5.0 |
| Feed/Rotation (mm) | 5 | 24 | 15 | 55 | 55 | 23 | 21 | 46 | 92 |
| Pitch | 1.5 | 1 | 0.625 | 1.375 | 1.375 | 1.2 | 1.1 | 0.6 | 1.2 |
| Kernel | Body filter (standard) | B30f | B40f | Stand | Stand | I30f | I31f/B70 | B31f | B31f |

**Supplementary Table S2.** List of radiomic features extracted via LASSO logistic regression analysis.

| Selected radiomic features | Note |
| --- | --- |
| **original series** |  |
| original_firstorder_RootMeanSquared |  |
| **wavelet series** |  |
| wavelet.LH_glcm_IverseVariance |  |
| wavelet.HL_glcm_IverseVariance |  |
| wavelet.HH_glcm_InverseVariance |  |
| wavelet.HH_glrlm_RunEntropy |  |
| wavelet.LL_firstorder_90Percentile | not considered due to high correlation with original_firstorder_RootMeanSquared |
| wavelet.LL_firstorder_TotalEnergy | not considered due to high correlation with original_firstorder_RootMeanSquared |
| wavelet.LL_glcm_MCC |  |

**Supplementary Table S3. (A)** The model performance in predicting L1 T-score and **(B)** the diagnostic accuracy of bone health^a^ between the Hounsfield model and three predictive models.

**(A)**

| Model | RMSE | MSE | MAE | Multiple R-square | Adjusted R-square | AIC | BIC |
| --- | --- | --- | --- | --- | --- | --- | --- |
| Hounsfield model | 0.982 | 0.965 | 0.777 | 0.412 | 0.411 | 1160.575 | 1172.638 |
| Model I: Clinical data | 1.120 | 1.254 | 0.867 | 0.236 | 0.229 | 1274.369 | 1298.495 |
| Model II: Radiomic data | 0.945 | 0.894 | 0.746 | 0.456 | 0.452 | 1132.860 | 1152.965 |
| Model III: Clinical + Radiomic data | 0.846 | 0.715 | 0.658 | 0.564 | 0.557 | 1049.164 | 1085.353 |

Abbreviations: RMSE, root mean square error; MSE, mean square error; MAE, mean absolute error; AIC, Akaike information criterion; BIC, Bayes information criterion.

**(B)**

| Predicted bone health | Hounsfield model | Model I: Clinical data | Model II: Radiomic data | Model III: Clinical + Radiomic data |
| --- | --- | --- | --- | --- |
| Sensitivity | 76.6% | 73.0% | 77.9% | 83.6% |
| Specificity | 71.0% | 61.3% | 72.0% | 74.2% |
| Total accuracy | 74.0% | 67.7% | 75.2% | 79.4% |

^a^ Bone health was determined based on L1 T-score, and was categorized as normal (T-score ≥ -1) and bone loss (T-score <-1).

Sensitivity (True positive rate) = Truly predicted bone loss / truly observed bone loss.
Specificity (True negative rate) = Truly predicted normal bone health / truly observed normal bone health.

Total accuracy = (True positive + True negative) / Total populations.
